# The Genome Sequence of ‘*Mycobacterium massiliense*’ Strain CIP 108297 Suggests the Independent Taxonomic Status of the *Mycobacterium abscessus* Complex at the Subspecies Level

**DOI:** 10.1371/journal.pone.0081560

**Published:** 2013-11-27

**Authors:** Yong-Joon Cho, Hana Yi, Jongsik Chun, Sang-Nae Cho, Charles L. Daley, Won-Jung Koh, Sung Jae Shin

**Affiliations:** 1 Chunlab, Inc., Seoul National University, Seoul, Korea; 2 Department of Public Health Sciences, Graduate School, Korea University, Seoul, Korea; 3 Korea University Guro Hospital, Korea University College of Medicine, Seoul, Korea; 4 Department of Microbiology and Institute for Immunology and Immunological Diseases, Yonsei University College of Medicine, Seoul, Korea; 5 Division of Mycobacterial and Respiratory Infections, Department of Medicine, National Jewish Health, Denver, Colorado, United States of America; 6 Division of Pulmonary and Critical Care Medicine, Department of Medicine, Samsung Medical Center, Sungkyunkwan University, School of Medicine, Seoul, Korea; National Institute of Infectious Diseases, Japan

## Abstract

Members of the *Mycobacterium abscessus* complex are rapidly growing mycobacteria that are emerging as human pathogens. The *M. abscessus* complex was previously composed of three species, namely *M. abscessus* *sensu stricto*, ‘*M. massiliense*’, and ‘*M. bolletii*’. In 2011, ‘*M. massiliense*’ and ‘*M. bolletii*’ were united and reclassified as a single subspecies within *M. abscessus*: *M. abscessus* subsp. *bolletii*. However, the placement of ‘*M. massiliense*’ within the boundary of *M. abscessus* subsp. *bolletii* remains highly controversial with regard to clinical aspects. In this study, we revisited the taxonomic status of members of the *M. abscessus* complex based on comparative analysis of the whole-genome sequences of 53 strains. The genome sequence of the previous type strain of ‘*Mycobacterium massiliense*’ (CIP 108297) was determined using next-generation sequencing. The genome tree based on average nucleotide identity (ANI) values supported the differentiation of ‘*M. bolletii*’ and ‘*M. massiliense*’ at the subspecies level. The genome tree also clearly illustrated that ‘*M. bolletii*’ and ‘*M. massiliense*’ form a distinct phylogenetic clade within the radiation of the *M. abscessus* complex. The genomic distances observed in this study suggest that the current *M. abscessus* subsp. *bolletii* taxon should be divided into two subspecies, *M*. *abscessus* subsp. *massiliense* subsp. nov. and *M*. *abscessus* subsp. *bolletii*, to correspondingly accommodate the previously known ‘M. massiliense’ and ‘*M*. *bolletii*’ strains.

## Introduction

Rapidly growing mycobacteria (RGM) are nontuberculous mycobacteria that cause a wide spectrum of human infections; the *Mycobacterium abscessus* complex is the most frequent group associated with pulmonary, skin, soft tissue, and bone diseases [1,2]. The *M*. *abscessus* complex is the most drug-resistant mycobacterial species known, and isolates are typically only susceptible *in vitro* to some parenteral agents (amikacin, cefoxitin, and imipenem) and macrolides (clarithromycin and azithromycin) [1,2], resulting in less than satisfactory treatment response rates [3,4]. Moreover, the number of cases of *M. abscessus* complex diseases has been reported to be increasing and have become emerging infectious diseases [3-9]. 

The *M*. *abscessus* complex has undergone many taxonomic changes since its first description in 1953 [10,11]. Because of the heterogeneity within the group, the *M. abscessus* complex (*M. abscessus* *sensu lato*) was divided into three species in 2006, namely *M. abscessus* *sensu stricto*, ‘*M. massiliense*’ [12], and ‘*M. bolletii*’ [13]. The three independent species were proposed primarily based on differences in the *rpoB* sequences and phenotypic patterns of the type strains [12,14]. However, the intraspecies-level phenotypic variation subsequently identified in clinical isolates rendered the three species phenotypically indistinguishable [15]. Thus, the differentiation of these three species has relied upon the sequencing of one or more housekeeping genes, such as *rpoB*, *hsp65*, and *secA*, although the emergence of isolates with interspecific composite patterns has led to conflicting identification results [16]. For example, isolates from recent Brazilian outbreaks display genetic characteristics consistent with either ‘*M. massiliense*’ or ‘*M. bolletii*’, depending on the housekeeping genes selected for the identification [15]. 

In 2011, *M. abscessus*, ‘*M. massiliense*’, and ‘*M. bolletii*’ were eventually united as a single species, *M. abscessus*, based on their overlapping phenotypic patterns, 100% 16S rRNA gene sequence identity, and >70% DNA-DNA hybridization values [17]. Simultaneously, two subspecies were proposed within this taxon based on the internal variability of the genotypes. The name *M. abscessus* subsp. *abscessus* was proposed to accommodate the *M. abscessus* *sensu stricto* strains, whereas the name *M. abscessus* subsp. *bolletii* was proposed to accommodate the previously known ‘*M. massiliense*’ and ‘*M. bolletii*’ strains [17]. The two subspecies can be distinguished genotypically by a single *Hae*III band difference in a PCR restriction analysis (PRA)-*hsp65* pattern [15]. In addition, the two subspecies share less than 96.6 and 98.7% sequence similarities in the 711-bp *rpoB* and 401-bp *hsp65* gene sequences, respectively [15]. 

However, the suitability of combining the ‘*M. massiliense*’ and ‘*M. bolletii*’ strains as a single subspecies, *M. abscessus* subsp. *bolletii*, remains a matter of debate [18], mainly because they are clinically different in terms of antibiotic susceptibility and treatment [14,19-22]. In fact, *M. abscessus* subsp. *abscessus* harbors an erythromycin ribosomal methylase gene, *erm* (41), and the presence of this gene is associated with inducible resistance to macrolides [23]. Indeed, inducible resistance to clarithromycin has been suggested as an explanation for the lack of efficacy of clarithromycin-based treatments against *M. abscessus* subsp. *abscessus* infection. By contrast, the ‘*M. massiliense*’ group exhibits marked susceptibility to clarithromycin, and inducible resistance to clarithromycin has not been observed during prolonged incubation [20]. Therefore, treatment response rates to clarithromycin-based antibiotic therapy are higher in patients with ‘*M. massiliense*’ group lung disease than those infected with *M. abscessus* subsp. *abscessus* [20]. However, unlike other RGM, the ‘*M. bolletii*’ group, a relatively rare pathogen at present [5,24-26], has proven to be highly resistant to clarithromycin [5,13,14,19]. 

Differential diagnosis of the subtypes within the *M. abscessus* complex has been strongly recommended to determine clinical significance and assist patient management [1]. However, the traditional molecular methods (e.g. PRA or *rpoB* and *hsp65* sequence analysis) occasionally results in the inaccurate discrimination of the complex [15,16]. In addition, the *M. abscessus* complex has undergone the taxonomic changes [11,15,17] and there appears to be a lack of consensus for the nomenclature of these taxa. Thus, the valid and acceptable taxonomic-designation of the *M. abscessus* complex is needed to be proposed. 

With the advent of next-generation sequencing, which permits the analysis of high-quality genome sequences and their comparison with other genomes in public databases, taxonomic studies are increasingly using average nucleotide identity (ANI) as a method of re-classification [27]. Accordingly, in this study, we sequenced the genome of ‘*Mycobacterium massiliense*’ strain CIP 108297 and revisited the taxonomic status of ‘*M. massiliense*’ and ‘*M. bolletii*’ based on the analysis of their whole-genome sequences to ascertain whether the two tested strains comprise a single subspecies status at the genomic level.

## Materials and Methods

### Genome sequencing

For the genome sequencing, strain CIP 108297, the previous type strain of ‘*M. massiliense*’, was obtained from CIP (Collection of Institut Pasteur, Paris, France), and genomic DNA was extracted with a Wizard genomic DNA purification kit (Promega, WI). The draft genome sequence of strain CIP 108297 was determined by 100 base-paired end sequencing reads using the Genome Analyzer IIx (Illumina, CA), which generated 20,301,763 reads that have Q30 over 79.9%. The mapped reads covered whole genome over 364-fold and 1.6% of total reads remained only as unmapped to the genome. The sequencing reads were assembled using the CLC genomics workbench 4.5 (CLCbio, Denmark) and CodonCode Aligner (CodonCode Co., MA). 

### Annotation and comparative genomics

Gene identification and annotation were achieved using the RAST server [28]. Orthologous gene prediction and comparative genomic analyses were conducted at the nucleotide and amino acid levels, as described previously [29]. In brief, a segment on a target contig homologous to a query ORF was identified using the BLASTN program, and this potentially homologous region was expanded in both directions by 1,000 bp. The nucleotide sequences of the query ORF and the selected target homologous region were then aligned using a pairwise global alignment algorithm [30], and the resulting matched region in the subject contig was extracted and saved as a homolog. For amino acid comparison, BLASTP comparison of query ORFs to subject ORFs was performed using only annotated genomes. Orthologs and paralogs were differentiated by reciprocal comparison. All genome sequences belonging to the genus *Mycobacterium* which compared each other in this study were obtained from NCBI microbial genome resources (http://www.ncbi.nlm.nih.gov/genome).

### ANI calculation & tree construction

The inter-genomic distances between two genome sequences were determined from fully or partially sequenced genomes using the average nucleotide identity (ANI) [31]. For a given pair of genomes, the query genome was cut into small pieces *in silico* (1020 bp), and the high-scoring segment pairs (HSPs) between the two genome comparison were determined using the BLAST algorithm [32,33]. The ANI was then calculated from the sets of HSPs, and its complement to 1 was considered for converting ANI into a distance. From this pairwise distance matrix, an ANI tree was constructed using the UPGMA (Unweighted Pair Group Method with Arithmetic Mean) clustering method. 

### Orthologous gene tree construction

Using the calculated gene-by-gene similarity, orthologous genes above a defined level of similarity were selected and then aligned using CLUSTALW [34]. The resulting multiple alignments were concatenated and used to construct a genome tree by the neighbor-joining method [35], as implemented in the MEGA program [36]. An evolutionary distance matrix for the neighbor-joining tree was generated according to the model of Jukes & Cantor [37]. The resultant tree topologies were evaluated by bootstrap analyses [38].

## Results

### Genome sequence

**Figure 1:**
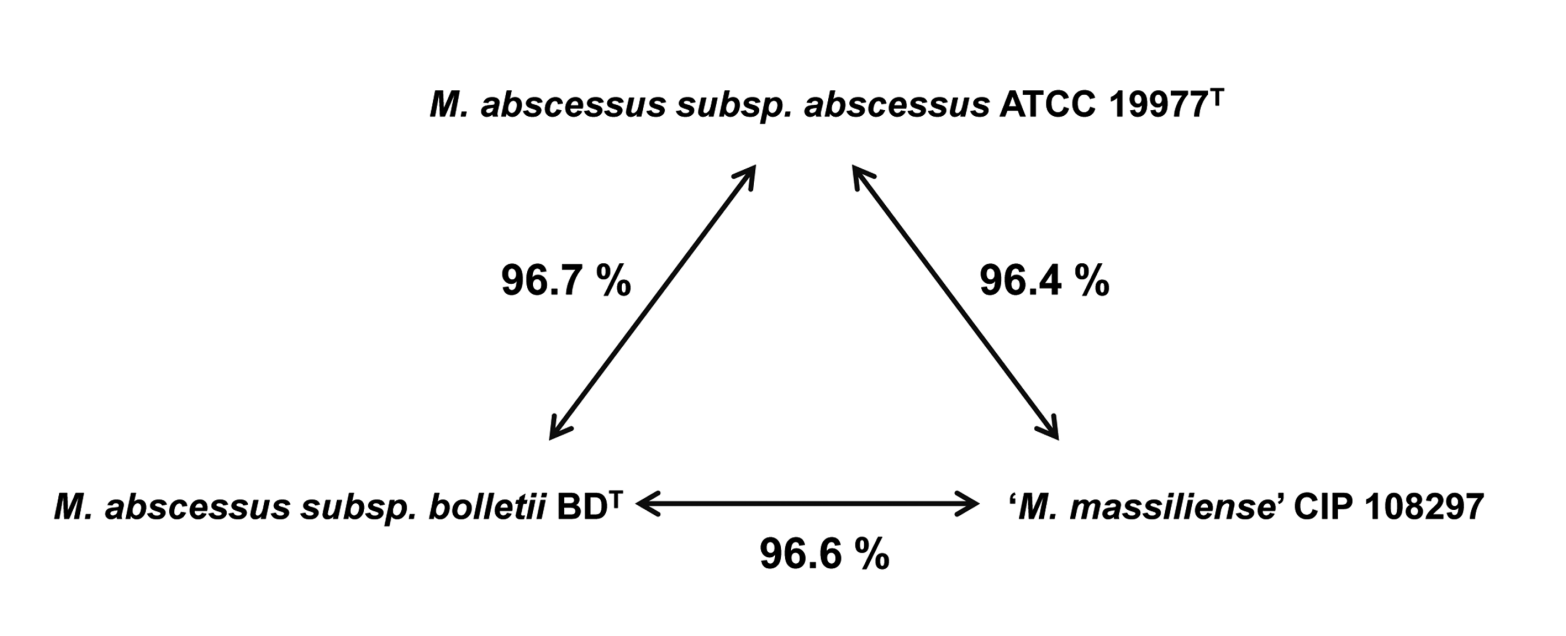
Genomic relatedness among the three representative strains based on genome sequences. The percent values represent the average nucleotide identity (ANI) values by pairwise alignment of genome stretches based on BLAST. The numbers shown are the average of the ANI value calculated in both directions.

The genome sequence of strain CIP 108297 was assembled into 18 contigs (>1 kb long) and deposited into GenBank under the project number PRJNA80701. The genome size was 4.97 Mb (excluding the gaps), with a G+C content of 64.1% and 4,828 predicted CDSs. *M. abscessus* subsp. *abscessus* ATCC 19977^T^ (PRJNA61613) [39] and *M. abscessus* subsp. *bolletii* BD^T^ (PRJNA73695) [40] were selected as representative strains, and their genomes were compared with the newly sequenced genome. The overall genomic characteristics of the representative strains were well positioned within the range of the genomes of the *M. abscessus* complex (Figure 1 and Table 1). 

**Table 1:** Genomic characteristics of representative strains belonging to the *Mycobacterium abscessus* complex.

	Genome size (bp)	%G+C	Contigs	CDSs	GenBank number	Reference
*M. abscessus* subsp. *abscessus* ATCC 19977**^*T*^**	5,090,491	64.1	2	4,941	PRJNA61613	(38)
*M. abscessus* subsp. *bolletii* BD**^*T*^**	5,048,007	64.1	22	4,923	PRJNA73695	(39)
‘*M. massiliense*’ CIP 108297	4,969,787	64.1	18	4,828	PRJNA80701	This study

### Genome tree

**Figure 2:**
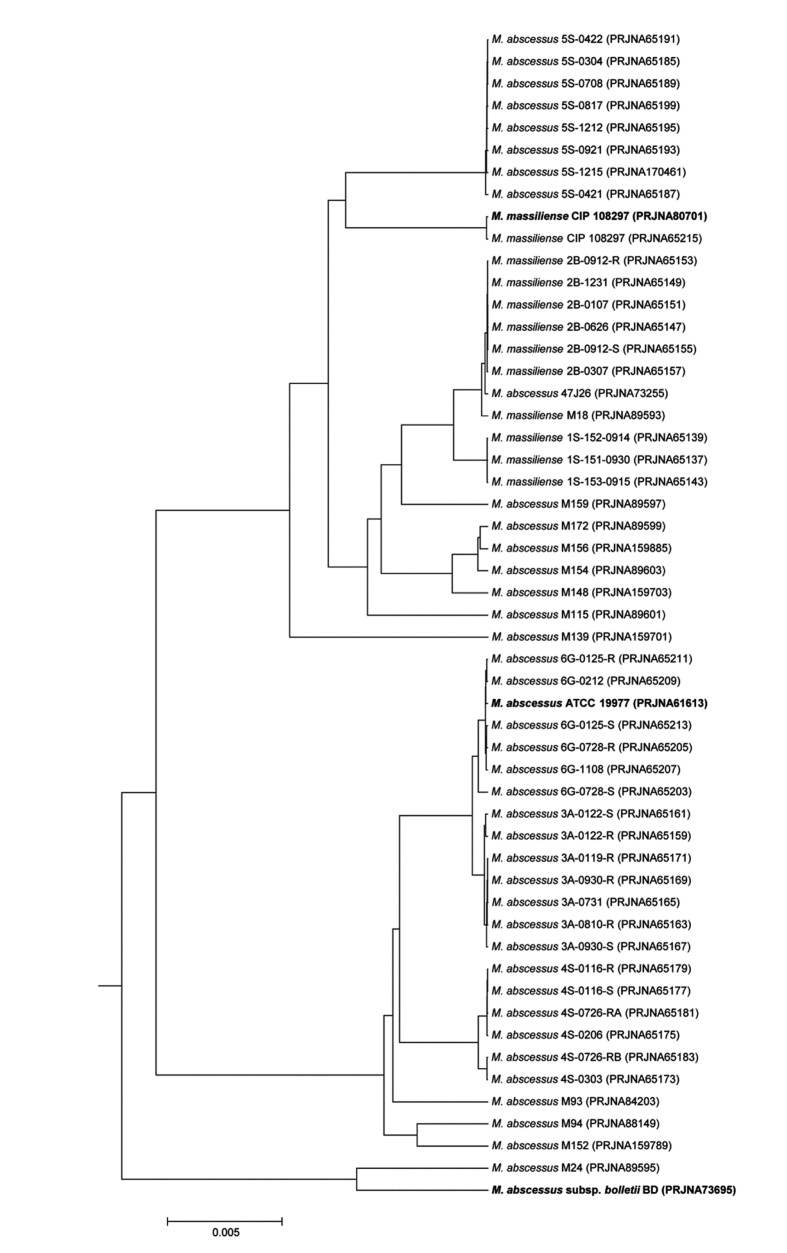
The genome tree based on ANI values showing the evolutionary relationships among 53 *Mycobacterium abscessus* genomes available in NCBI microbial genome resources (http://www.ncbi.nlm.nih.gov/genome). To convert the ANI into a distance, its complement to 1 was taken. From this pairwise distance matrix, an ANI tree was constructed using the UPGMA clustering method.

The ANI genome tree, a dendrogram based on ANI values showing evolutionary relationships, was generated using 53 *M. abscessus* genomes available in NCBI microbial genome resources (http://www.ncbi.nlm.nih.gov/genome) (Figure 2). The three representative strains, *M. abscessus* subsp. *abscessus* ATCC 19977^T^, *M. abscessus* subsp. *bolletii* BD^T^ and ‘*Mycobacterium massiliense*’ strain CIP 108297, are located at three distinct *M. abscessus* clusters, indicating the clear grouping at the *M. abscessus* species level.

### Comparative analysis of *Mycobacterium abscessus* genomes and orthologous gene trees

**Figure 3:**
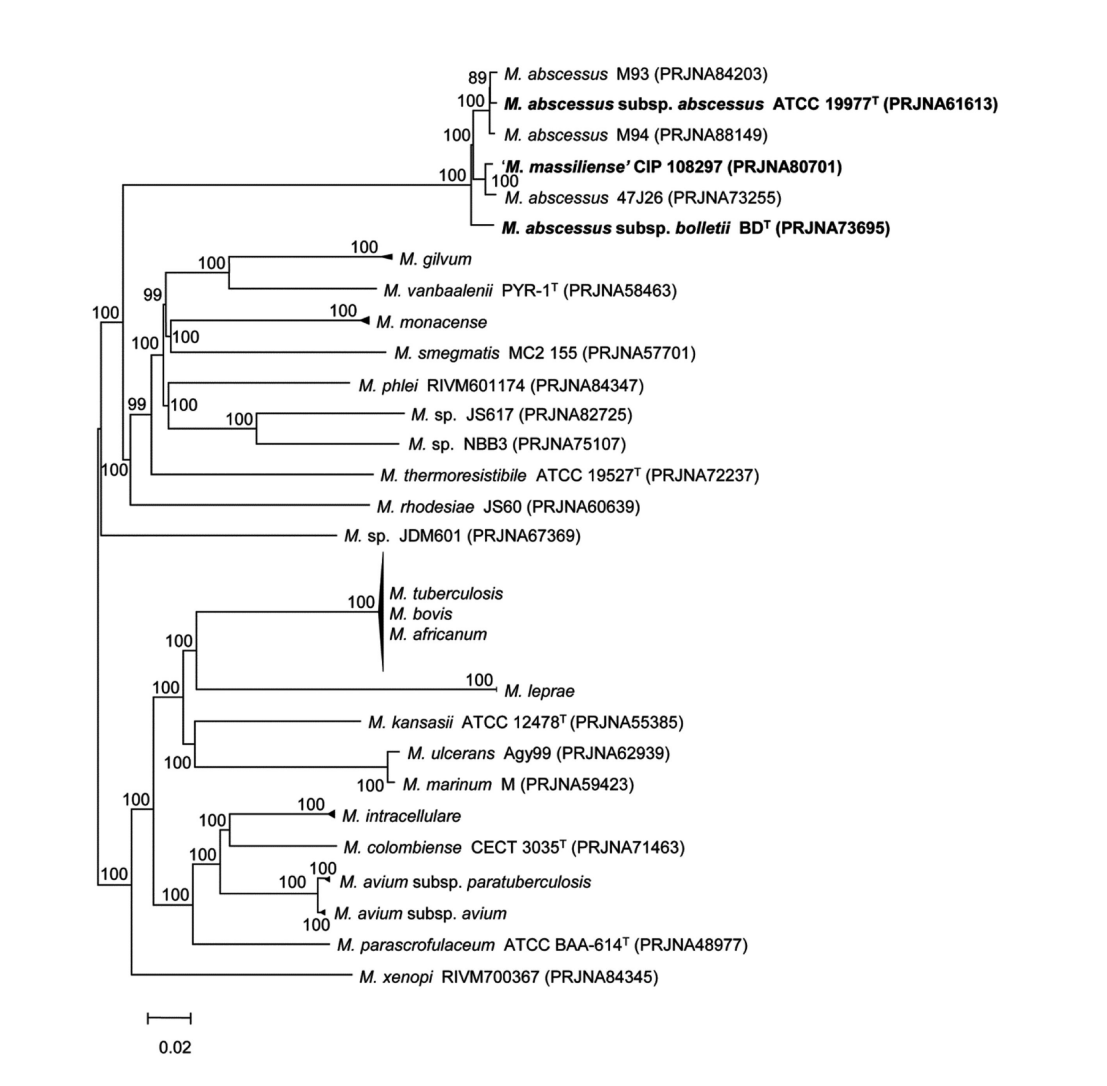
The genome tree based on 262 highly conserved orthologous genes of 81 annotated genomes of *Mycobacterium*. The evolutionary distance matrix was calculated using the Jukes & Cantor model, and the tree was constructed using the neighbor-joining method. Each node number represents the percentage of bootstrap support (>70%) from 1,000 resampled datasets. The bar represents 0.02 substitutions per site.

**Figure 4:**
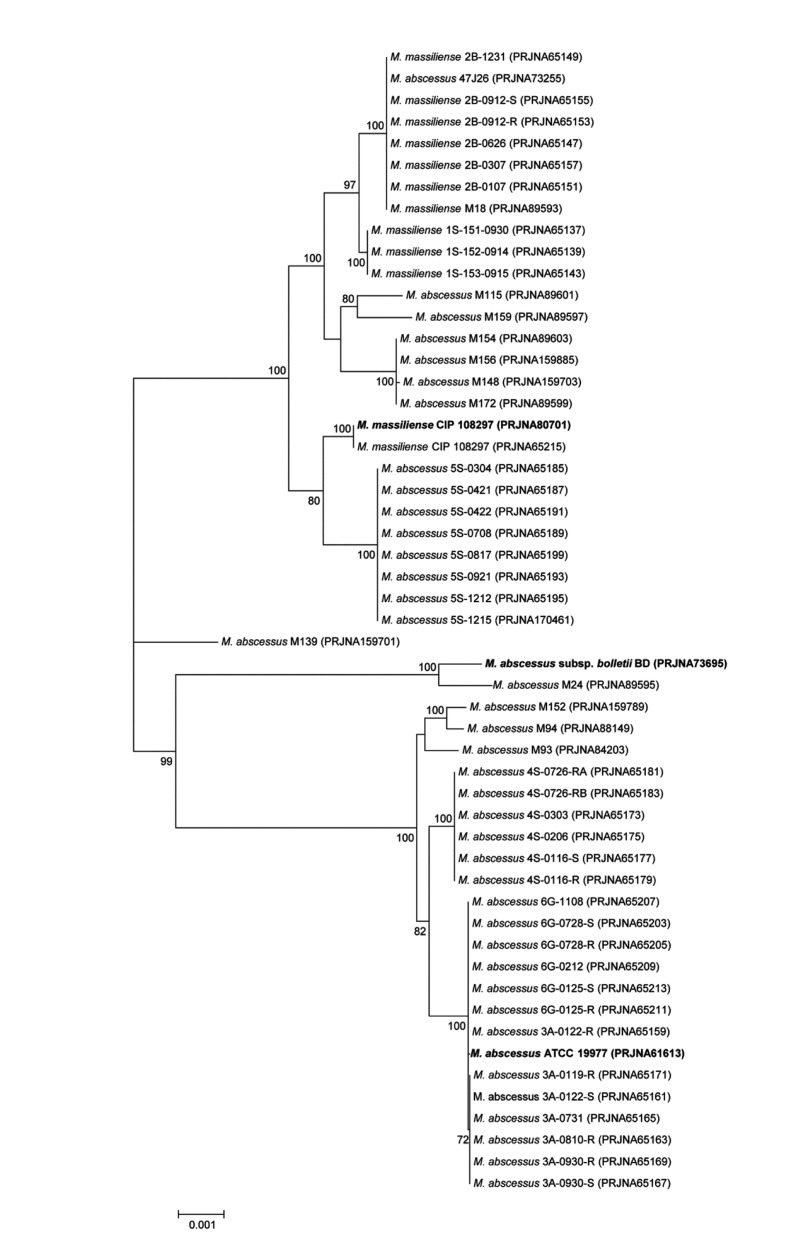
The phylogenetic tree based on 11 concatenated housekeeping genes conserved in the *Mycobacterium abscessus* group. Each node number represents the percentage of bootstrap support (>70%) from 1,000 resampled datasets. The gene sequences of the *argH, cya, gdhA, glpK, gnd, murC, pgm, pknA, pta, purH*, and *rpoB* genes were obtained from 53 genome-sequenced *Mycobacterium abscessus* strains, and the sequences were aligned and concatenated (18,893 bp in length). The bar represents 0.002 substitutions per site.

**Figure 5:**
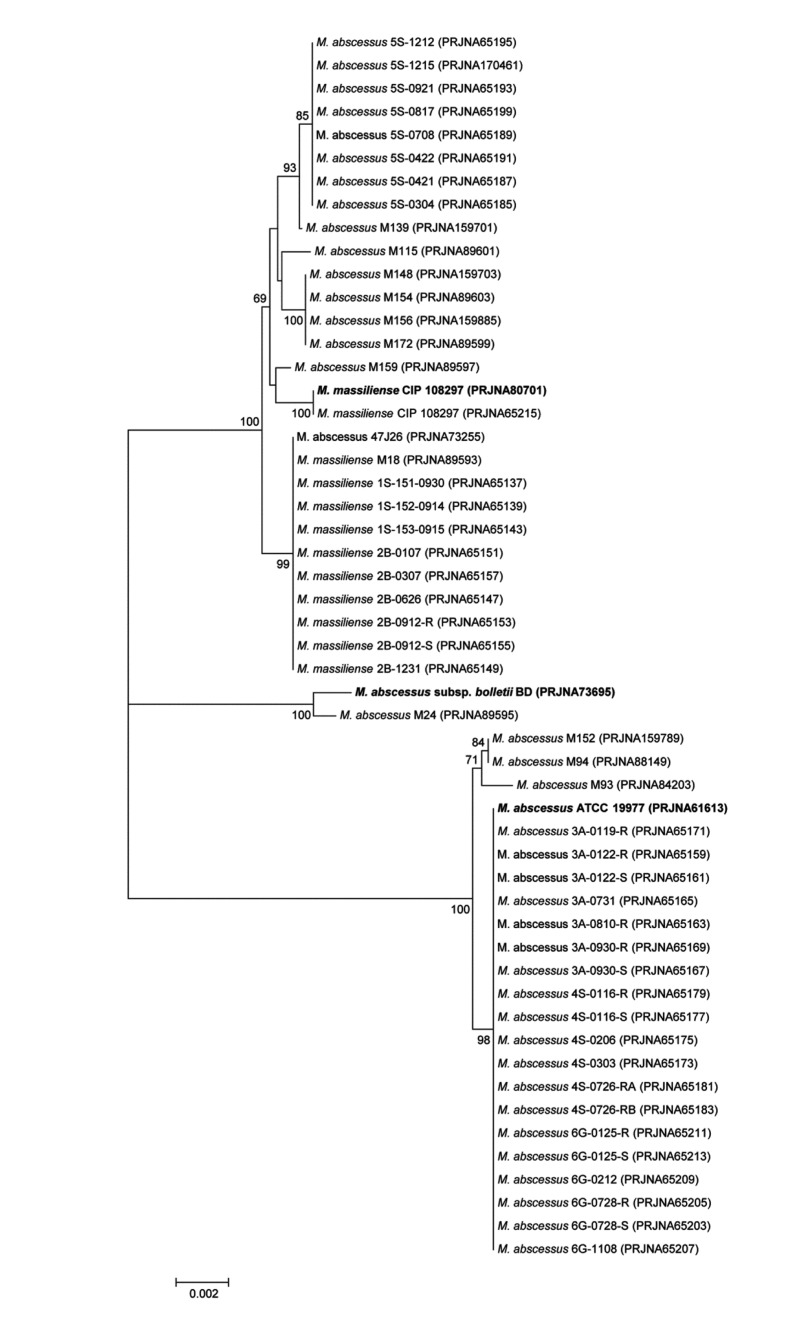
The *rpoB* gene-based neighbor-joining tree showing the relationships among 53 genome-sequenced *Mycobacterium abscessus* strains. Each node number represents the percentage of bootstrap support (>70%) from 1,000 resampled datasets. The bar represents 0.001 substitutions per site.

A comparative analysis at genus level from 81 annotated *Mycobacterium* genomes available in NCBI microbial genome resources (http://www.ncbi.nlm.nih.gov/genome) was performed. All ORFs from one genome was compared to those from other genome, and bidirectional best hits (BBHs) were made for each pair of 81 strains using BLASTP to find orthologs [41,42]. A set of highly conserved orthologous ORFs (262 genes in total, 237,940 bp) showing greater than 50% amino acid sequence similarity to *Mycobacterium abscessus* subsp. *abscessus* ATCC 19977^T^ (PRJNA61613) was selected to represent highly conserved proteins of the genus *Mycobacterium* [43]. An orthologous gene tree was built using aligned amino acid sequences, demonstrating that the three representative strains clustered among the other *Mycobacterium* strains (Figure 3). In addition, 11 housekeeping genes (*argH, cya, gdhA, glpK, gnd, murC, pgm, pknA, pta, purH*, and *rpoB*, 18,893 bp) were selected for the species-level comparison from a previous study [44] of 53 *Mycobacterium abscessus* genomes (Figure 4). Their nucleotide sequences were aligned using CLUSTALW and the phylogenetic tree was drawn. Finally, phylogenetic tree was built using the *rpoB* single-gene nucleotide sequence (Figure 5) because the *rpoB* sequence has been widely used as a taxonomic-classification marker [45,46]. The overall tree structure was similar, with the representative strains forming their own clusters (Figures 4 and 5). In addition, a comparative BLASTP analysis based on the three representative strains revealed group-specific genes among the representative strains (see Information S1). 

## Discussion

In this study, we evaluated the current taxonomic status of *M. abscessus* and its subspecies using a similarity analysis based on the single gene, multilocus gene, several hundreds of orthologous gene, and whole-genome levels. This species has recently been regrouped as follows: *M. abscessus* subspecies *bolletii* by uniting *M. bolletii* and *M. massiliense*, and *M. abscessus* subspecies *abscessus* [17]. The classification of these bacteria has been based on polymorphisms in various target genes [47]; however, the introduction of whole-genome sequencing has increased the availability of genetic information to facilitate the identification of organisms. 

The ANI value between a given pair of genomes has been recognized as a simple and effective method to reflect the degree of evolutionary distance between the compared genomes [31,32]. Comparative studies have reported that a value of 94-96% identity represents a DNA-DNA hybridization boundary of 70%, the gold standard for categorizing a bacterial species under the current prokaryotic species concept [31,32,48,49]. The ANI values among the three genomes representing the three groups of the *M. abscessus* complex were all above the cut-off value for species demarcation, clearly indicating that the three strains belong to the same species (see Information S2). Within this species boundary, the genomic distances of the three strains from one another were equivalent (Figure 1). Moreover, a greater difference between strain CIP 108297 and *M. abscessus* subsp. *abscessus* ATCC 19977^T^ (3.5% of difference) was observed than between the two type strains of the two established subspecies (3.3%). This relatively high genomic distance strongly suggests the independence of the ‘*M. massiliense*’ group from the ‘*M. bolletii*’ group at the subspecies level. 

This degree of genetic difference between the ‘*M. massiliense*’ and ‘*M. bolletii*’ strains is also supported by an ANI-based genome tree and a phylogenetic tree using orthologous gene alignment. The three representative strains form three distinct phylogenetic clades within the radiation of *M. abscessus*, indicating the existence of three independent subspecies within the species (Figures 2 and 3). Of note, many strains in public databases that are named *M. massiliense* and *M. abscessus* are mixed in the same cluster of the genome tree, obviously reflecting the need to clarify the *M. abscessus* subsp. *abscessus* clade and revise the current taxonomic status (Figure 2).

Previous studies using single or multilocus sequencing approaches (MLSAs) have yielded inconsistent results [5,15,44]. Indeed, discrepancies between an *rpoB*-based gene tree and MLSA sequence identification results have been reported, with results indicating low divergence values among these groups [44]. In the present study, phylogenetic trees based on single or several genes appeared to be less powerful for the discrimination of the boundary strains (Figures 4 and 5). *M. abscessus* M139 belonged to the ‘*M. massiliense*’ group in the *rpoB* single-gene tree (Figure 5) yet was not clustered with any representative group in the MLSA sequence tree (Figure 4). It has been proposed that horizontal gene transfer between the *M. abscessus*, *M. massiliense*, and *M. bolletii* groups causes inconsistency in subspecies clustering [44], and our ANI-based genome tree definitely included *M. abscessus* M139 in the ‘*M. massiliense*’ group (Figure 2). In addition, the single-gene and MLSA sequence trees in this study showed three distinct, well-clustered groups, but the currently available *M. abscessus* whole-genome sequences are biased toward specific sampling sites. Indeed, if the sequences from various clinical strains are added, the single-gene or MLSA sequence tree would show an ambiguous shape, as reported previously. To identify other classification criteria, 25 representative group-specific genes were selected as molecular identification candidates by a comparative genome analysis (see Information S1). For more clear identification, all homologs which can be found in other *Mycobacterium* genus genomes were excluded from the candidates. Recently, Shallom et al. proposed by an array based genomic approach that subspecies-level separation is a more reasonable taxonomic identification of the *M. abscessus* group [50]. These results are consistent with our current study, indicating that the subspecies-level identification of the *M. abscessus* group should include three taxa: *M. abscessus* subsp. *abscessus*, *M. abscessus* subsp. *massiliense*, and *M. abscessus* subsp. *bolletii*. 

Based on the low genomic relatedness (96.5% ANI against the other two representative strains) and the distinct phylogenetic clade discovered in this study, it is evident that ‘*M. massiliense*’ is not affiliated within *M. abscessus* subsp. *bolletii*. Thus, we propose a novel subspecies within *M. abscessus* to accommodate the previously known ‘*M. massiliense*’, namely *M. abscessus* subsp. *massiliense* subsp. nov. Strain CIP 108297 is the type strain of the newly suggested subspecies.

Taken together, although the genetic characteristics responsible for the different phenotypes among the representative groups were not identified, the genomic content that clearly divides these strains into three clusters and their differential drug susceptibility patterns indicate the need to reevaluate the taxonomic status of *M. abscessus* and classify representative groups into three subspecies. Increasing whole-genome data would facilitate the differentiation of genetic factors for the molecular differentiation of boundary strains with insufficient diversity.

## Supporting Information

Information S1 **Group-specific genes for *M. abscessus* subsp. *abscessus* ATCC 19977^T^ (PRJNA61613), *M. abscessus* subsp. *bolletii* BD^T^ (PRJNA73695), and ‘*M. massiliense*’ CIP 108297 (PRJNA80701) in 53 *M. abscessus* genomes.**
(XLS)

Information S2 **Average nucleotide identity (ANI) values for the pairs of genomes evaluated in this study.**
(DOCX)
