# Supplementary material for: The Genome Sequence of ‘Mycobacterium massiliense’ Strain CIP 108297 Suggests the Independent Taxonomic Status of the Mycobacterium abscessus Complex at the Subspecies Level
Source: PLoS One. 2013 Nov 27;8(11):e81560. doi: 10.1371/journal.pone.0081560 (PMC3842311; doi:10.1371/journal.pone.0081560)
Supplement: Information S2 — Average nucleotide identity (ANI) values for the pairs of genomes evaluated in this study. (DOCX) [file pone.0081560.s002.docx]

**Table S2.** Average nucleotide identity (ANI) values for the pairs of genomes evaluated in this study.

|  | ATCC 19977^T^ | BD^T^ | CIP 108297 |
| --- | --- | --- | --- |
| *M. abscessus* subsp. *abscessus* ATCC 19977^T^ | - | 96.70 | 96.24 |
| *M. abscessus* subsp. *bolletii* BD^T^ | 96.64 | - | 96.42 |
| ‘*M. massiliense’* CIP 108297 | 96.48 | 96.70 | - |
